# Mature Human Bone Marrow Plasma Cells Secrete More IgG than Early-Minted Blood Antibody-Secreting Cells

**DOI:** 10.21203/rs.3.rs-6585905/v1

**Published:** 2025-06-27

**Authors:** Doan C. Nguyen, Ian T. Hentenaar, Monica Cabrera-Mora, Shuya Kyu, Andrea Morrison-Porter, Natalie S. Haddad, Joel Andrews, Danielle Roberts, Sagar Lonial, Ignacio Sanz, F. Eun-Hyung Lee

**Affiliations:** 1 Division of Pulmonary, Allergy, Critical Care, and Sleep Medicine, Department of Medicine, Emory University, Atlanta, GA, United States;; 2 Department of Hematology and Medical Oncology, Winship Cancer Institute, Emory University, Atlanta, GA, United States;; 3 Division of Rheumatology, Department of Medicine, Emory University, Atlanta, GA, United States;; 4 Lowance Center for Human Immunology, Emory University, Atlanta, GA, United States

## Abstract

Plasma cells are known antibody-secreting factories with immunoglobulin (Ig) transcripts that increase as the cell matures into a long-lived plasma cell (LLPC) in the bone marrow (BM). Whether the Ig secretion rates among human antibody-secreting cells (ASC) are homogeneous or BM LLPC are capable of secreting more antibodies per cell compared to early-minted blood ASC remain unclear. Here, we use bulk and single cell cultures in a novel *in vitro* BM mimetic survival system to measure the IgG secretion rates of human ASC. We find that the mature BM ASC produce more IgG molecules per cell compared to immature, early-minted blood ASC. Furthermore, these blood ASC can mature into LLPC phenotypes in culture, and we show that ASC on day 7 secrete more IgG per cell than the input ASC from day 0. Thus, as human ASC mature, they increase the number of Ig transcripts and result in higher Ig secretion. These results also demonstrate that the mature ASC in the BM have higher Ig secretion rates compared to early-minted blood ASC.

## Introduction

Human antibody-secreting cells (ASC) are now well recognized to be heterogeneous, consisting of early-minted ASC in the blood and intermediate and mature plasma cells in the bone marrow (BM)^1–3^. In the blood, immature ASC can also be quite heterogeneous^4^ while in the BM, ASC can be transcriptionally classified as short-lived, intermediate, and long-lived plasma cells (LLPC) which have the potential to survive indefinitely^5–7^. Although difficult to study *in vivo*, the model of human ASC maturation into a LLPC was initially described with the use of a novel *in vitro* BM mimetic system^8,9^. For clarity, the term ASC is used to refer to all Ig-secreting cells, which include early-minted blood ASC (plasmablasts)^3^ and mature ASC known as plasma cells that can contain the LLPC subset.

As a protein secreting factory, a human ASC can produce massive amounts of immunoglobulins (Ig): 100–10,000 molecules per cell per second or 2–220 pg/cell/day (pg/c/d)^10–14^. Studies reported a wide range of Ig secretion rates with some as high as 3,334 pg/c/d^15^, while ASC differentiated *in vitro* from human B cells secrete less at 20–140 pg/c/d^16,17^, and malignant myeloma cells have even lower rates^18^. These Ig secretion rates were thought to be influenced by both extrinsic and intrinsic factors^9,19^ since mature ASC have expansion of subcellular secretory network (the Golgi and ER) and organelles important in metabolism (mitochondrial mass) which prepare the cells to specialize in Ig secretion^9,19^. In addition, we found increasing Ig transcripts from blood to BM ASC, with the most mature LLPC subset containing the highest number of Ig transcripts^20^. Whether these differences ultimately translate into higher Ig secretion rates remain unexplored.

Since ASC rapidly die *ex vivo*, we use a cell-free specialized culture system derived from factors within the human BM microniche that enables survival of ASC to measure Ig secretion rates^8^. Since this system also mimics the BM microniche, we can follow the maturation process of blood ASC into a mature BM ASC phenotype^9^. Here, we show that mature BM ASC secrete more Ig per cell compared to early-minted blood ASC, illustrating the heterogeneity of Ig secretion rates. As blood ASC mature in this BM mimetic system, they begin to secrete more Ig over time, demonstrating the importance of the maturation process for increased Ig secretion.

## Results

### Mature ASC have higher IgG secretion rates.

**Fig. 1.**
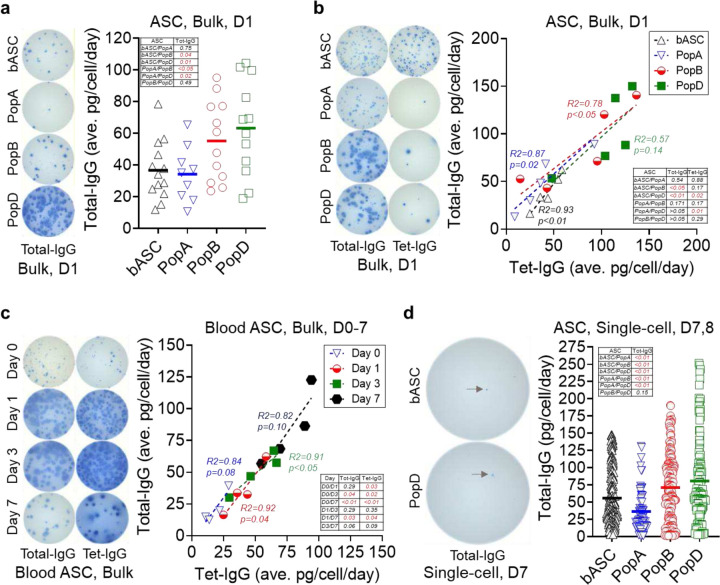
Mature ASC have higher IgG secretion rates by bulk and single-cell interrogation. (**a**) Average total IgG secretion rates by blood ASC and BM ASC maintained in the BM mimetic bulk cultures for 1 day. (*Left*) Representative ELISpot scanned images; the input ASC numbers (seeded at day 0): ~600, 480, 800, and 1080, for blood ASC, PopA, PopB, and PopD, respectively. (*Right*) Data generated from 13, 9, 12, and 12 independent biological experiments for blood ASC, PopA, PopB, and PopD, respectively; each symbol represents one experiment. (**b**) Average total IgG and Tet-IgG secretion rates by blood ASC and BM ASC maintained in the BM mimetic bulk cultures for 1 day. (*Left*) Representative ELISpot scanned images; the input ASC numbers (seeded at day 0): ~1000, 7117, 918, and 941 (total IgG) and ~3000, 56936, 5508, and 2823 (Tet-IgG) for blood ASC, PopA, PopB, and PopD, respectively. (*Right*) Data generated from 5, 5, 5, and 5 independent biological experiments for blood ASC, PopA, PopB, and PopD, respectively; each symbol represents one experiment (per each subset). (**c**) Average total IgG and Tet-IgG secretion rates by blood ASC maintained in the BM mimetic bulk cultures for up to seven days. (*Left*) Representative ELISpot scanned images; the input ASC numbers (seeded at day 0): ~1800, 1800, 1800, and 900 (total IgG) and ~3600, 3600, 3600, and 1800 (Tet-IgG) for 0, 1, 3, and 7 days, respectively. (*Right*) Data generated from four independent biological experiments; each symbol represents one experiment (per each timepoint). (**d**) Total IgG secretion rates by blood ASC and BM ASC maintained in the BM mimetic single-cell cultures for 7–8 days. (*Left*) Representative ELISpot scanned images. (*Right*) Data generated from 248, 72, 176, and 110 individual cells; each symbol represents one cell. In (**a**-**d**), inserted is a table of *p* values calculated with Student’s t-test (two-tailed unpaired *t-test*) in Excel (Microsoft). In (**b**, **c**), R and *p* values calculated from simple linear regression analysis in GraphPad Prism (GraphPad Software). In (**c**), blood ASC day 1 data reproduced from four out of five blood ASC experiments shown in (**b**). bASC, early-minted blood ASC; Tet, tetanus.

We first interrogated production of total IgG in healthy human ASC *ex vivo* from the blood and the BM. In one-day bulk cultures of blood ASC, the average IgG secretion rate is 37±19 pg/c/d, which is similar to PopA (34±17 pg/c/d), but significantly lower than PopB (55±26 pg/c/d) or PopD (63±30 pg/c/d) (Fig. 1a). While secretion rates by blood ASC or PopA are significantly lower than PopB or PopD, no significant differences exist between PopB vs PopD. Thus, IgG secretion rates increase from blood ASC and early BM ASC (PopA) to mature BM ASC (PopB and PopD). This finding is consistent with high Ig transcript abundance in PopD, the most mature BM ASC subset^20^.

### Myeloma cells have lower IgG secretion rates relative to healthy blood ASC.

To understand secretion rates of healthy vs diseased plasma cells, we evaluated a myeloma cell line, ARH-77. As shown previously, the size of the IgG ELISpots of this myeloma cell line was much smaller compared to that of healthy blood ASC or BM ASC, suggesting lower secretion rates^3^. Corroborating this finding, we show ARH-77 secrete on average 19±11 pg/c/d (Fig. s1) which is less than the rates from blood or BM ASC. Appreciating that primary myeloma cells and myeloma cell lines are also very heterogeneous, it is likely myeloma cell secretion rates may be quite variable. Nonetheless, for this particular established myeloma cell line, secretion rates are lower than those of healthy human ASC.

### Mature ASC have higher vaccine-specific IgG secretion rates.

Consistent with the step-wise maturation concept^20^, a similar course of progressive maturity can occur with antigen-specific ASC^21^. We next examined the blood ASC from adults on day 6–7 after Tdap vaccination, which is the peak of the vaccine responses, as well as the BM ASC from adults 5–10 years after the Tdap vaccine. We evaluated if average Tdap vaccine-specific (Tet-) IgG secretion rates differ from total IgG secretion rates in blood and BM ASC in one-day bulk cultures^22^. As expected^5,22^, the frequencies of Tet-IgG ASC are 35±15% of the total IgG ASC in the blood days after vaccination, compared to the Tet-IgG ASC frequences of 0.3±0.2%, 1.2±0.6%, and 2.4±1.1% of total IgG PopA, PopB, and PopD, respectively, in the BM years after the vaccination. We observe blood ASC and PopA are the lowest secretors: average total IgG of 39±18 pg/c/d or 50±30 pg/c/d, and average Tet-IgG of 43±14 pg/c/d or 41±31 pg/c/d for blood ASC or PopA, respectively (Fig. 1b). For mature BM ASC, PopB and PopD secrete substantially higher total IgG (average of 86±43 pg/c/d and 101±41 pg/c/d, respectively) and Tet-IgG (average of 78±49 pg/c/d and 105±34 pg/c/d, respectively). As shown, there are differences between average total IgG secretion rates of blood ASC vs PopB and blood ASC vs PopD, and between average antigen-specific (Tet-) IgG secretion rates of blood ASC vs PopD and PopA vs PopD. Thus, significant differences in secretion rates are based on the maturity of the ASC and not necessarily the antigen of interest. For example, if vaccine-specific IgG rates are different from total IgG secretion rates, there may be heterogeneity of ASC based on the specificity of the Ig the cell produced. However, the IgG secretion rates for total and vaccine-specific were similar based on the ASC subsets, demonstrating that the secretion rate differences are based on ASC maturity.

### Blood ASC after in vitro maturation have higher IgG secretion rates.

We next assessed if the same immature blood ASC could increase IgG secretion rates after *in vitro* maturation. We measured blood ASC total IgG and Tet-IgG secretion rates at day 0, 1, 3, and 7 in culture. Total IgG and Tet-IgG gradually accumulated in the supernatants (Fig. s2), suggesting cultured blood ASC maintain functionality overtime. Assuming individual blood ASC produce the same Ig amount each day and have equal survival rates within the assessing period, we observe a progressive increase in average total IgG secretion rates: from 22±12 pg/c/d at day 0 to 36±19 pg/c/d at day 1, then 50±16 pg/c/d at day 3, and 84±29 pg/c/d at day 7 (Fig. 1c). While no significant difference exists between average total IgG secretion rates for day 0 vs day 1, day 1 vs day 3, and day 3 vs day 7, there is significance between day 0 vs day 3, day 0 vs day 7, and day 1vs day 7. We also see a similar pattern of progressive increase in average Tet-IgG secretion rates: 19±8 pg/c/d, 41±14 pg/c/d, 52±17 pg/c/d, and 77±18 pg/c/d at day 0, day 1, day 3, and day 7, respectively. Thus, the same blood ASC has higher average total IgG and Tet-IgG secretion rates following *in vitro* maturation. These kinetics experiments of human blood ASC maturation are the first to clearly illustrate these differences.

### Mature ASC have higher IgG secretion rates on a single cell basis.

We next studied if total IgG secretion rates generated from bulk cultures are corroborated by single cell cultures after 7–8 days. We see that on a single cell basis, in blood ASC, total IgG secretion rate is 56±39 pg/c/d, while for BM ASC, total IgG secretion rates are the lowest in PopA (37±30 pg/c/d) and higher in PopB (71±48 pg/c/d) and PopD (81±64 pg/c/d), with no significant difference between PopB vs PopD (Fig. 1d). Thus, similar to bulk cultures, mature ASC have higher total IgG secretion rates on a single cell basis. Although not statistically significant, single blood ASC surviving in 7-day single cell cultures have a greater variability of IgG secretion rates compared to 1-day bulk cultures, most likely due to ongoing maturation in the cultures even at the single cell level.

We then investigated total IgG and influenza-specific (Flu-) IgG secretion rates in single-sorted blood ASC maintained *in vitro*. Of 192 single-cells, total IgG and Flu-IgG are detected in 165 (~86%) and 114 (~59%), respectively, indicating high plating efficiency. Again, we note similar secretion rates for total IgG and Flu-IgG. We also show a wide range of total IgG and Flu-IgG secretion rates (3–148 pg/c/d and 12–142 pg/c/d, respectively) with average total IgG secretion rates of 48±42 pg/c/d and Flu-IgG secretion rates of 35±40 pg/c/d (Fig. s3). Again, the single culture system shows an increase in total IgG and vaccine-specific IgG secretion rates of blood ASC as they undergo ongoing maturation in the BM mimetic cultures, but the wide ranges may reflect early apoptosis of blood ASC.

### Mature ASC have higher IgG secretion rates by direct single cell visualization.

**Fig. 2.**
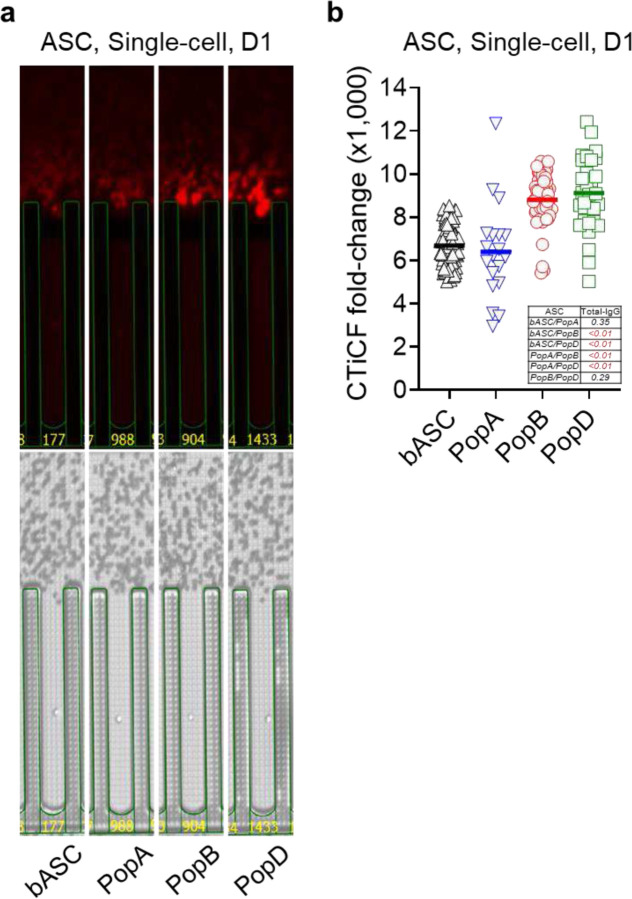
Mature ASC have higher IgG secretion rates by single cell visualization. (**a**) Representative image series displaying ongoing IgG secretion by a single blood ASC and BM ASC maintained in the BM mimetic single-cell cultures on-chip for one day. (*Lower*) Bright-field image series for direct visualization of a single cell in a nanopen. (*Upper*) The act of ongoing IgG secretion into the channel developed as fluorescent halos (“bloom”; captured at the end of an 80-minute cycle). (**b**) CTiCF fold-changes as an indirect comparison of total IgG secretion rates among four ASC subsets. Data generated from 97, 19, 56, and 35 individual in-pen cells for blood ASC, PopA, PopB, and PopD, respectively; each symbol represents one cell. Inserted is a table of *p* values calculated with Student’s t-test (two-tailed unpaired t-test) in Excel (Microsoft). All four ASC subsets were run on the same chips (for CTiCF comparison). bASC, early-minted blood ASC.

To overcome apoptosis and ongoing maturation in 7-day single cultures, we directly interrogated single ASC secretion rates. Total IgG bloom is captured (Fig. 2a) and semi-quantitatively calculated as CTiCF fold-changes from the bachground^3^. We show PopB and PopD secrete more IgG per cell compared to blood ASC or PopA, with average CTiCF fold-change by blood ASC, PopA, PopB, or PopD is 6,687, 6,397, 8,809, or 9,120, respectively (Fig. 2b). Significant fold-change differences (*p*<0.01) exist between blood ASC vs PopB, blood ASC vs PopD, PopA vs PopB, and PopA vs PopD, but not (*p*>0.29) between blood ASC vs PopA or PopB vs PopD. Thus, blood ASC or PopA consistently produces less IgG per cell than PopB or PopD, suggesting increased Ig transcripts^20^ lead to increased Ig secretion.

## Discussion

In this study, we show that total IgG and vaccine-specific IgG secretion rates are higher in mature BM plasma cells (PopB and PopD) compared to immature blood ASC and BM PopA, which may include new arrivals and cells near death. As a fraction of blood ASC migrate to BM^21,23,24^, the majority of these new arrivals die while some undergo progressive maturation^5,9,21,24^. Early studies showed transcriptional differences between BM plasma cells compared to earlier non-BM plasma cells^25,26^. Furthermore, single-cell RNAseq revealed heterogeneity of the human BM plasma cell pool and offered a trajectory analysis with a step-wise maturation process from new arrivals to a *bona fide* LLPC^20^. The findings of this study provide significant insights into the functional heterogeneity of human ASC and their maturation-dependent Ig secretion capacities.

We demonstrate that ASC maturation is accompanied by both elevated Ig transcript levels^20^ and increased Ig secretion capacity, highlighting the enhanced antibody-producing potential of mature BM plasma cells. Our results contradict previous thoughts that blood ASC had required rapid Ig production with high Ig secretion rates in response to acute infections, and that mature BM plasma cells have lower and slower secretion rates to sustain long-term immunity. Our findings align with the model that in the BM niche, ASC maturation undergo transcriptional and/or post-transcriptional adaptations such as elevated Ig transcript levels in LLPC^20^ to optimize their role as antibody-secreting factories.

A key contribution of this study is the use of a novel *in vitro* BM mimetic survival system^8^, which allowed us to track the maturation of ASC^9,20^ and quantify their IgG secretion dynamics over time. The finding that early-minted blood ASC cultured in this system for 7 days secreted more IgG per cell than their day 0 counterparts suggests that the factors in the BM microenvironment play a critical role in driving functional maturation. This increase in Ig secretion capacity may reflect upregulation of Ig transcripts, enhanced ER function, or improved secretory pathway efficiency as ASC mature^27^. These results underscore the importance of the BM niche in supporting not only plasma cell survival but also their functional specialization. This *in vitro* BM mimetic survival system offers a new tool for studying human plasma cell biology, including ASC maturation *in vitro*.

The observed differences in IgG secretion rates between early-minted blood ASC and mature BM plasma cells have implications for understanding humoral immunity. Higher secretion rates in BM plasma cells likely contribute to the sustained antibody production required for long-term immune protection, such as in response to vaccination or infection^7,22^. This efficiency of BM plasma cells to secrete nearly twice as much antibody per cell suggests prioritizing higher efficiency and fewer LLPC needed to achieve serologic protection. Conversely, the lower secretion rates of early-minted blood ASC may reflect their transient role in acute immune responses. These findings raise questions about the molecular mechanisms underlying the transition from early ASC to mature plasma cells, especially LLPC, including potential epigenetic or signaling pathways that regulate Ig secretion capacity.

While our study provides robust evidence for maturation-dependent increases in IgG secretion, certain limitations warrant consideration. The *in vitro* BM mimetic system, although effective in supporting ASC maturation, may not fully recapitulate the complexity of the *in vivo* BM niche, including interactions with cytokines or other immune cells. Additionally, our focus on IgG secretion leaves open questions about whether similar trends apply to other Ig isotypes, such as IgA or IgM, which may be relevant in specific immune contexts. Future studies are needed to investigate the translational potential of modulating ASC maturation to enhance antibody responses in clinical settings, such as vaccine or immunotherapy.

In conclusion, our study highlights the functional specialization of human ASC as they mature into BM LLPC, with significant increases in per-cell IgG secretion rates. Our results are consistent with increased Ig transcripts of mature BM plasma cells^20^ which results in higher Ig secretion rates. These findings advance our understanding of plasma cell biology and emphasize the critical role of the BM niche in optimizing antibody production. As LLPC are the basis of a successful vaccine^7^, our findings emphasize the role of ongoing ASC maturation in the BM and highlight the importance of filling the BM LLPC compartment^22^ for more stable and durable immunity. Further exploration of the molecular and environmental factors driving these changes will be essential for harnessing the full potential of ASC in therapeutic applications.

## Methods

### Human subjects.

We enrolled 69 healthy adults (aged 23–65 years) for peripheral blood samples collected 6–7 days after vaccination with influenza, tetanus (Tdap), hepatitis A, hepatitis B, shingles, HPV, or COVID-19. We also obtained 32 BM aspirates from healthy adults (aged 21–68 years, with 13 men (41%) and 19 women (59%)). Samples were collected during July 2014-March 2024. All research was approved by the Emory University Institutional Review Board and written informed consent was obtained from all subjects.

### ASC purification and a human myeloma cell line.

Mononuclear cells from peripheral blood and BM aspirate samples were isolated, enriched, and stained as previously described^5,8^. Cells were bulk- or single cell-sorted on a BD FACSAria II as^3,5,8^: blood ASC (IgD^−^CD27^hi^CD38^hi^) (Fig. s4a), BM PopA (CD19^+^IgD^−^D38^hi^CD138^−^), BM PopB (CD19^+^IgD^−^CD38^hi^CD138^+^), and BM PopD (CD19^−^IgD^−^CD38^hi^CD138^+^) (Fig. s4b). Among these BM ASC subsets, PopA represent immature subset, PopD (LLPC) are the most mature one, and PopB are more heterogeneous or an intermediate phenotype^5,20,22^. The human myeloma cell line, ARH-77, was purchased (ATCC).

### In vitro BM mimetic cultures.

ASC were cultured for one day or up to 7–8 days in the BM mimetic system, which consists of the BM mesenchymal stromal cell (MSC) survival medium and in hypoxia at 37°C supplemented with APRIL^8^. ARH-77 cells were handled as per recommendations^3^.

### ELISpots, ELISA, and multiplex bead binding assays (MBBA).

We quantified total and vaccine-specific IgG ASC using ELISpots, as previously described^3,8,22^ (Fig. s5). We measured secreted IgG by ELISA or MBBA, as previously described^22^. As the amount of total IgG secreted by a single ASC in one day was below the detection limit by our assays, we maintained single-sorted ASC *in vitro* for 7–8 days. Average IgG concentrations were calculated based on the number of spots and the concentrations of secreted IgG (assuming equal secretion rates both per cell and per day). Also, although blood ASC could increase Ig secretion rates with maturation in the cultures, we measured the cumulative total IgG amount in the 7-day culture from a single blood ASC and BM PopA, PopB, and PopD, and divided by 7 (days). For vaccine-specific IgG capturing, tetanus toxoid Clostridium tetani (Calbiochem) or quadrivalent influenza vaccine 2015–2016 (Fluarix Quadrivalent Influenza Vaccine 2015–2016 Formula; GSK Biologicals) was used. For relative quantitation of the concentrations of total IgG or antigen-specific IgG, standard curves were generated using purified human IgG (ChromePure human IgG, JacksonImmuno Research Laboratories) or monoclonal antibody (mAb) standards of anti-tetanus toxin mAb (clone #TetE3; The Native Antigen Company).

### On-chip single-cell culture, in-channel IgG capture, and fluorescence semi-quantitation.

To overcome apoptosis and ongoing maturation in the 7-day single ASC cultures, we directly assess single ASC secretion rates using a novel microfluidic system to visualize IgG secretion *ex vivo* with high precision^3^. Single ASC visualization was performed using the Lightning system (Bruker Cellular Analysis)^3^. The instrument captures individual ASC in the act of Ig secretion^3^ which develops as fluorescent halos (“blooms”). We loaded blood ASC and BM ASC on the Lightning platform and directly visualized IgG production from a single primary ASC *ex vivo* within 30 minutes^3,8^. Semi-quantitation of relative fluorescent signal intensity of blooms was performed using ImageJ (NIH)^3,28^. Specifically, we calculated the relative corrected total in-channel fluorescence intensity (CTiCF) across an in-channel region of interest by subtracting out the mean fluorescence of background (Reference) readings as follows: CTiCF = Integrated density – (Area of selected region x Mean fluorescence of background readings)^28^.

### Statistics.

Statistical differences and correlation were evaluated using Excel (Microsoft) and GraphPad Prism (GraphPad Software). A *p* value of ≤0.05 was considered significance.

## Supplementary Material

1 Supplementary Fig. s1-s5.
(**s1**) Average total IgG secretion rates by ARH-77 cells maintained in the *in vitro* BM mimetic bulk cultures for one day. (*Left*) Representative ELISpot scanned images; the input ASC number (seeded at day 0): ~2000. (*Right*) Data generated from 8 independent biological experiments; each symbol represents one experiment.
(**s2**) Cumulative total IgG and Tet-IgG by early-minted blood ASC maintained in bulk cultures for up to seven days. Data generated from four independent biological experiments; each symbol represents one experiment (per each timepoint). Inserted is a table of *p* values calculated with Student’s t-test (two-tailed unpaired *t-test*) in Excel (Microsoft).
(**s3**) Total IgG and Flu-IgG secretion rates by blood ASC maintained in single-cell cultures for 7–8 days. Data generated from 192 individual cells; each symbol represents one cell. R and *p* values calculated from simple linear regression analysis in GraphPad Prism (GraphPad Software) of data generated from 114 cells (out of individual 192 cells) positive for both total IgG and/or Flu-IgG. Flu, influenza.
(**s4**) General FACS gating strategy used for sorting blood ASC and BM ASC. (**a**) PBMC or (**b**) BMMC were first gated for lymphocytes, singlets, and viable cells (based on FSC/SSC and Live/Death properties). CD3 and CD14 were then used as dump markers to capture CD19+ and CD19^−^ B cell populations. (**a**) Subsequent sub-gating using CD38 vs CD27 on the IgD^−^ fraction (of CD19^+^ population) allows for sorting for blood ASC (IgD^−^CD27^hi^CD38^hi^) (**b**) Subsequent sub-gating from CD19^+^ population on the IgD^−^ fraction (vs CD27) and using CD138 versus CD38 allowed for breaking down BM ASC populations into three subsets of interest: PopA (CD19^+^CD38^hi^CD138^−^), PopB (CD19^+^CD38^hi^CD138^+^), and PopD (LLPC; CD19^−^CD38^hi^CD138^+^).
(**s5**) Summary of the techniques and experimental designs for detection of total IgG, Tet-IgG, and Flu-IgG ASC, as well as IgG secreted in the culture supernatants by ELISpots and the Lightning platform, as well as by ELISA and MBBA, respectively.

## Data Availability

There are no restrictions on the availability of experimental data from and of unique materials used in this study. All the data generated and/or analyzed in this study are available from the corresponding author. All unique materials used are readily available from the corresponding author and Emory University. Source data are provided with this paper.
