# Supplementary figures and images for: Mature Human Bone Marrow Plasma Cells Secrete More IgG than Early-Minted Blood Antibody-Secreting Cells
Source: Res Sq. 2025 Jun 27:rs.3.rs-6585905. Preprint. [Version 1] doi: 10.21203/rs.3.rs-6585905/v1 (PMC12270214; doi:10.21203/rs.3.rs-6585905/v1)

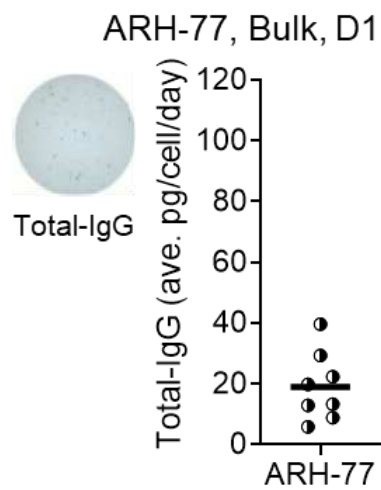

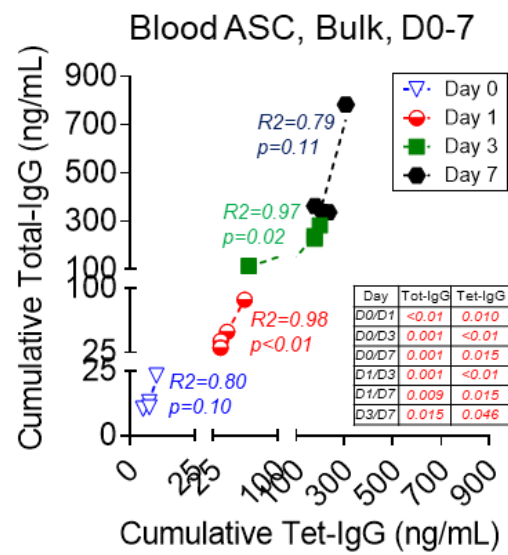

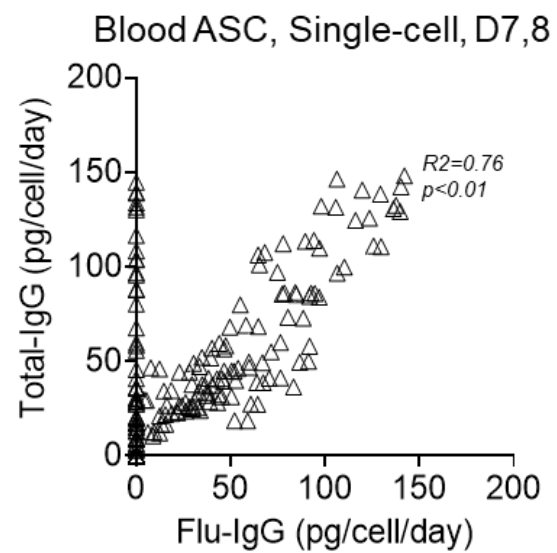

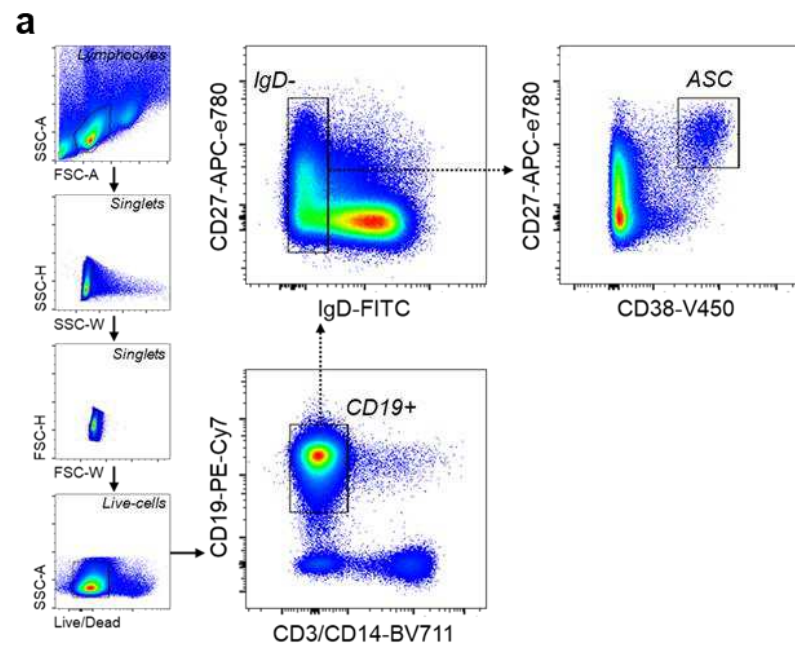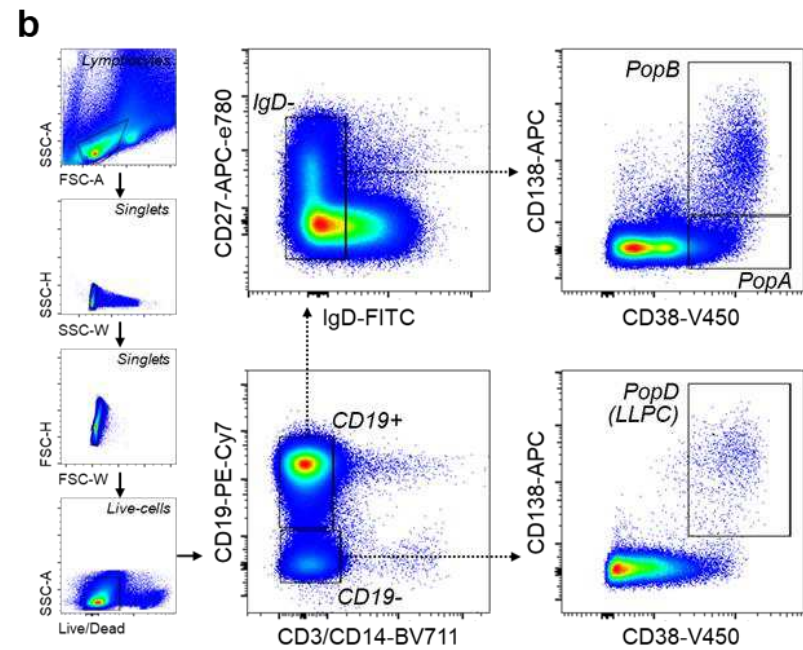

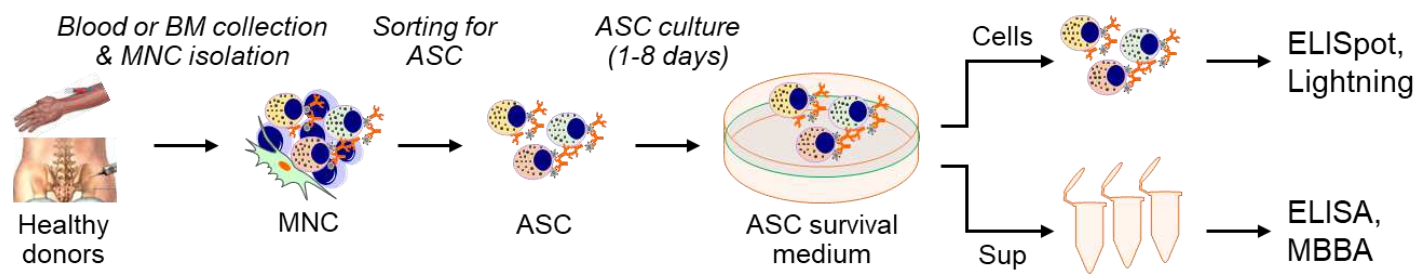

Supplement: 1 — Supplementary Fig. s1-s5. (s1) Average total IgG secretion rates by ARH-77 cells maintained in the in vitro BM mimetic bulk cultures for one day. (Left) Representative ELISpot scanned images; the input ASC number (seeded at day 0): ~2000. (Right) Data generated from 8 independent biological experiments; each symbol represents one experiment. (s2) Cumulative total IgG and Tet-IgG by early-minted blood ASC maintained in bulk cultures for up to seven days. Data generated from four independent biological experiments; each symbol represents one experiment (per each timepoint). Inserted is a table of p values calculated with Student’s t-test (two-tailed unpaired t-test) in Excel (Microsoft). (s3) Total IgG and Flu-IgG secretion rates by blood ASC maintained in single-cell cultures for 7–8 days. Data generated from 192 individual cells; each symbol represents one cell. R and p values calculated from simple linear regression analysis in GraphPad Prism (GraphPad Software) of data generated from 114 cells (out of individual 192 cells) positive for both total IgG and/or Flu-IgG. Flu, influenza. (s4) General FACS gating strategy used for sorting blood ASC and BM ASC. (a) PBMC or (b) BMMC were first gated for lymphocytes, singlets, and viable cells (based on FSC/SSC and Live/Death properties). CD3 and CD14 were then used as dump markers to capture CD19+ and CD19− B cell populations. (a) Subsequent sub-gating using CD38 vs CD27 on the IgD− fraction (of CD19+ population) allows for sorting for blood ASC (IgD−CD27hiCD38hi) (b) Subsequent sub-gating from CD19+ population on the IgD− fraction (vs CD27) and using CD138 versus CD38 allowed for breaking down BM ASC populations into three subsets of interest: PopA (CD19+CD38hiCD138−), PopB (CD19+CD38hiCD138+), and PopD (LLPC; CD19−CD38hiCD138+). (s5) Summary of the techniques and experimental designs for detection of total IgG, Tet-IgG, and Flu-IgG ASC, as well as IgG secreted in the culture supernatants by ELISpots and the Lightning pla [file NIHPPRS6585905V1-supplement-1.pdf]
